# Psychiatric Treatment Conducted via Telemedicine Versus In-Person Modality in Posttraumatic Stress Disorder, Mood Disorders, and Anxiety Disorders: Systematic Review and Meta-Analysis

**DOI:** 10.2196/44790

**Published:** 2023-07-05

**Authors:** Ali Abbas Shaker, Stephen F Austin, Ole Jakob Storebø, Julie Perrine Schaug, Alaa Ayad, John Aasted Sørensen, Kristine Tarp, Henrik Bechmann, Erik Simonsen

**Affiliations:** 1 Psychiatric Department, Region Zealand Psychiatry, Psychiatric Research Unit Slagelse Denmark; 2 Department of Clinical Medicine, University of Copenhagen Copenhagen Denmark; 3 Department of Psychology, University of Southern Denmark Faculty of Health Sciences Odense Denmark; 4 Department of Engineering Technology and Didactics, Research unit: AI, Mathematics and Software, Technical University of Denmark Ballerup Denmark; 5 Research Unit for Digital Psychiatry, Mental Health Services in the Region of Southern Denmark Odense Denmark; 6 Department of Clinical Research, University of Southern Denmark Odense Denmark; 7 Mental Health Services East, Copenhagen University Hospital – Psychiatry Region Zealand Roskilde Denmark

**Keywords:** telemedicine, telepsychiatry, video consultation, mobile health, mHealth, eHealth, COVID-19, synchronous technology, anxiety, psychiatry, patient satisfaction, depression, posttraumatic stress disorder, PTSD

## Abstract

**Background:**

Telemedicine has played a vital role in providing psychiatric treatment to patients during the rapid transition of services during the COVID-19 pandemic. Furthermore, the use of telemedicine is expected to expand within the psychiatric field. The efficacy of telemedicine is well described in scientific literature. However, there is a need for a comprehensive quantitative review that analyzes and considers the different clinical outcomes and psychiatric diagnoses.

**Objective:**

This paper aimed to assess whether individual psychiatric outpatient treatment for posttraumatic stress disorder, mood disorders, and anxiety disorders in adults using telemedicine is equivalent to in-person treatment.

**Methods:**

A systematic search of randomized controlled trials was conducted using recognized databases for this review. Overall, 4 outcomes were assessed: treatment efficacy, levels of patient satisfaction, working alliance, and attrition rate. The inverse-variance method was used to summarize the effect size for each outcome.

**Results:**

A total of 7414 records were identified, and 20 trials were included in the systematic review and meta-analysis. The trials included posttraumatic stress disorder (9 trials), depressive disorder (6 trials), a mix of different disorders (4 trials), and general anxiety disorder (1 trial). Overall, the analyses yielded evidence that telemedicine is comparable with in-person treatment regarding treatment efficacy (standardized mean difference −0.01, 95% CI −0.12 to 0.09; *P*=.84; *I*^2^=19%, 17 trials, n=1814), patient satisfaction mean difference (−0.66, 95% CI −1.60 to 0.28; *P*=.17; *I*^2^=44%, 6 trials, n=591), and attrition rates (risk ratio 1.07, 95% CI 0.94-1.21; *P*=.32; *I*^2^=0%, 20 trials, n=2804). The results also indicated that the working alliance between telemedicine and in-person modalities was comparable, but the heterogeneity was substantial to considerable (mean difference 0.95, 95% CI −0.47 to 2.38; *P*=.19; *I*^2^=75%, 6 trials, n=539).

**Conclusions:**

This meta-analysis provided new knowledge on individual telemedicine interventions that were considered equivalent to in-person treatment regarding efficacy, patient satisfaction, working alliance, and attrition rates across diagnoses. The certainty of the evidence regarding efficacy was rated as moderate. Furthermore, high-quality randomized controlled trials are needed to strengthen the evidence base for treatment provided via telemedicine in psychiatry, particularly for personality disorders and a range of anxiety disorders where there is a lack of studies. Individual patient data meta-analysis is suggested for future studies to personalize telemedicine.

**Trial Registration:**

PROSPERO International Prospective Register of Systematic Reviews CRD42021256357; https://www.crd.york.ac.uk/prospero/display_record.php?RecordID=256357

## Introduction

### Background

During the last 2 decades, there has been increasing interest in and publication of research studies addressing the effect of telemedicine on psychiatric patients [1-5]. Research has highlighted several potential advantages of using telemedicine in mental health services. Some of the most apparent advantages of integrating telemedicine in mental health services are enabling clinicians to reach out to patients living in rural areas and patients with mental health difficulties who find it challenging to attend treatment in person [1,2]. However, several studies have also reported challenges regarding the use of telemedicine for mental health services, including concerns about establishing a good patient-therapist alliance and the underuse of telemedicine by clinicians in resource-constrained clinics [3-5].

The experience of COVID-19 has placed an increased focus on the provision of interventions using telemedicine. This unique world situation, coupled with continual advances in technology, means that a regular synthesis of evidence for psychiatric interventions using telemedicine is warranted [6-10].

In recent years, several meta-analyses have compared the efficacy of psychiatric treatment provided using telemedicine with in-person treatment [11-13]. Drago et al [11] reviewed the evidence of psychiatric counseling (but not specific psychiatric or psychotherapeutic interventions) using telemedicine compared with in-person treatment. They included 24 randomized controlled trials (RCTs) primarily for posttraumatic stress disorder (PTSD) and major depression and found no difference in treatment effects between the 2 modalities. Their review did not examine the satisfaction, alliance, or attrition rates between the 2 modes of treatment. Batastini et al [12] conducted a large meta-analysis with broad inclusion criteria and combined data from a variety of study designs (RCTs and within subjects), reported outcomes (observer rated and self-report), and treatment format (individual and group). Their analysis included 43 studies and found that treatment effects were largely comparable between telemedicine and in-person modalities; however, they did not examine satisfaction, alliance, or attrition rates between the 2 modes of treatment. Giovanetti et al [13] focused exclusively on comparing psychotherapeutic interventions for depression. They included 11 RCTs and found that telemedicine-based psychotherapy had comparable efficacy with in-person psychotherapy [13]. They also found no differences in the attrition rates between the 2 modalities in patients diagnosed with depression.

### Objective

This meta-analysis builds on the results of previous reviews by addressing some of the deficiencies of earlier meta-analyses and providing a comprehensive and updated overview of the evidence for telemedicine in psychiatric settings. Thus, the primary aim of this systematic review and meta-analysis is to examine whether individual psychiatric outpatient interventions for adults using telemedicine are equivalent to the in-person format regarding treatment efficacy. As part of this comprehensive meta-analysis comparing the treatment effects between telemedicine and in person, we examined different diagnostic disorders and analyzed a range of moderators. Second, the meta-analysis addressed several gaps in current scientific research using standard and valid measures to examine the satisfaction, working alliance, and attrition rates between telemedicine and in-person modalities across a range of psychiatric diagnoses.

## Methods

### Overview

The methods section of this systematic review and meta-analysis is described in a published peer-reviewed protocol [14]. This systematic review was conducted according to the PRISMA (Preferred Reporting Items for Systematic Reviews and Meta-Analyses) guidelines [15]. The PRISMA checklist can be found on the web (Multimedia Appendix 1).

### Registration and Protocol

This systematic review was registered in PROSPERO (CRD42021256357). A peer-reviewed protocol has been published for this study. First, a post hoc analysis was conducted to evaluate attrition outcomes based on diagnosis (PTSD and Depression). Second, subgroup analysis for the moderators, “settings,” and “vulnerable populations” were poorly described in the included studies, and subgroup analysis for these moderators was therefore not applicable. Third, the title names have been adjusted. Apart from the listed amendments, no significant amendments were made compared with the published protocol.

### Inclusion Criteria

The eligibility criteria were based and restricted on the type of study, population, intervention, comparator, and outcomes of the studies.

#### Types of Studies

RCTs were considered.

#### Types of Participants

The participants were (1) adults (aged >18 years), (2) receiving individual psychiatric outpatient treatment, and (3) diagnosed with PTSD, mood disorders, anxiety, or personality disorders according to both the American Psychiatric Association’s Diagnostic and Statistical Manual of Mental Disorders III-V and the World Health Organization’s International Statistical Classification of Diseases 9 or 10. Participants with comorbid diagnoses were also included, with the exception of those diagnoses covered in the exclusion criteria.

#### Types of Interventions

Individual treatment through synchronous real-time video consultations in outpatient settings. Treatment was defined as an intervention that involved psychotherapy, pharmacological treatment, or psychoeducation.

#### Types of Comparators or Controls

The comparator was individual treatment in person with the same active treatment that the intervention group (telemedicine) received.

#### Types of Outcomes

The primary outcome was studies that assessed psychopathology (efficacy) after using a mental health service. The secondary outcomes of interest were (1) patient satisfaction, (2) working alliance, and (3) attrition rate.

### Exclusion Criteria

The following criteria were considered the reason for exclusion:

- Participant aged <18 years
- Group therapy
- Different psychotherapeutic (treatments) approaches used in the telemedicine and in-person modalities
- Trials involving populations primarily treating psychotic disorders, mental retardation, bipolar disorder, alcohol abuse, and substance use disorders
- Trials using asynchronous communication systems as an intervention (eg, emails and static websites without video function) and telephones with only audio function as an intervention

### Information Sources and Search Strategy

The first step in the systematic review was a comprehensive search in electronic databases. The database search strategy was developed using input from the project team. A search was conducted for studies published between 1967 and October 2022.

The following databases were used: MEDLINE (PubMed interface, 1986 onward), APA PsycINFO (Ovid interface, 1967 onward), Embase (Ovid interface, 1974 onward), Web of Science (Clarivate interface, 2001 onward), and CINAHL (EBSCOhost interface, 1981 onward).

Medical Subject Headings (MeSH) and text words related to the search terms “psychiatry” and “telemedicine” were used to develop the search string in MEDLINE.

Examples of MeSH and text words related to the term “psychiatry” included: (“Psychiatry”[MeSH Terms] or “Mental Disorders”[MeSH Terms] or “Mental Health Services”[MeSH Terms]) and (“mental health counseling”[Title/Abstract] or “mental health care”[Title/Abstract] or “psychiatric home care”[Title/Abstract] or “psychiatric outpatient*”[Title/Abstract]).

Examples of MeSH and text words related to the term “telemedicine” included: (“Telemedicine”[MeSH Terms] or “Videoconferencing”[MeSH Terms] or “Remote Consultation”[MeSH Terms] and “telecare”[Title/Abstract] or “teleconsultation*”[Title/Abstract] or “telemedic*”[Title/Abstract] or “telepsychiatr*”) Both search terms “psychiatry” and “telemedicine” were combined with (AND).

Specific syntax and subject headings were subsequently adapted individually to the different databases.

No language or date restrictions were implemented in the search process. Owing to the preliminary search’s unmanageable results (>20.000 hits), the highly sensitive search strategy filters of Cochrane identifying randomized trials were applied. Unpublished studies were not sought.

The second step in the search strategy was a manual literature search to identify additional primary studies for systematic review. The third step involved scanning the reference lists of the included studies or relevant reviews identified in the first and second steps, respectively.

### Data Management

Records from the literature search were exported to the reference manager Endnote X9 (Clarivate Analytics) [16]. From Endnote, records were exported to Covidence (Veritas Health Innovation), a web app tool that facilitates collaboration among review team members during the study selection and data extraction process [17]. Data extracted in Covidence were exported to RevMan 5.4 (Cochrane Collaboration) for data analysis [18].

### Selection Process

AAS and AA were responsible for the selection process. The 2 authors independently screened the titles and abstracts of the records in Covidence to identify potentially eligible records. The second step involved screening full-text reports to assess whether the reports met the eligibility criteria. Three authors (pairwise) were responsible for the second step (AAS, SFA, or JPS). Disagreements in the full-text screening process were resolved through discussions between the authors. A fourth reviewer, OJS, was consulted in case of continued disagreement despite discussion. The selection process was documented in the PRISMA flow diagram, including reasons for exclusion. Interrater reliability was measured using Cohen κ coefficient for the title and abstract screening and full-text review processes.

### Data Collection Process

AAS, SFA, and JPS were responsible for the data collection process. Data extraction was performed independently by 2 authors using a standardized electronic data extraction form in Covidence. The data extraction form was pilot tested on 5 reports, and the reviewers met and discussed the form before starting the review. Disagreements in the data collection process were resolved through discussions between the authors. A third reviewer (OJS) was consulted when disagreements could not be resolved between the independent authors. If multiple reports of the same study were encountered, data from all reports were extracted into a single data collection form in Covidence [19]. Missing data were obtained by contacting and requesting these data from the study authors.

### Data Items

We extracted the following data items for each study: (1) study characteristics (authors, author contact details, aim of the study, trial design, location, trial size, sample size calculation, year of publication, and country); (2) population characteristics (remote or rural area or urban, country, diagnosis or condition, mean age, and sex); (3) intervention or control (internet connection speed, bandwidth, therapy type, number of consultation sessions, and duration of consultation); and (4) clinical outcome (assessment tools, psychopathology [efficacy outcome], patient satisfaction, working alliance, and attrition rate). When reported in the studies, we collected data from the intention-to-treat analysis; otherwise, we collected data from the per-protocol analysis.

### Outcomes and Prioritization

The primary outcome was efficacy, as assessed by clinician or patient-rated scales. As we expected that different assessment tools had been used for measuring the primary outcome, we prioritized clinician-rated scales over patient-rated scales, should both be available.

The secondary outcomes were (1) patient satisfaction, (2) working alliance, and (3) attrition rate. The patient satisfaction measure was restricted to the Client Satisfaction Questionnaire-8 (CSQ-8) [20], and the working alliance was restricted to the Working Alliance Inventory-Client version (WAI-C) [21]. The attrition rate was defined as the proportion of individuals who withdrew after being randomized to a modality to the total number of participants randomized to a modality.

### Risk of Bias in Individual Studies

Authors AAS, SFA, and JPS performed (pairwise) the risk of bias (quality) assessment for the primary outcome (treatment efficacy) in each individual study, using the revised Cochrane risk-of-bias tool for randomized trials (RoB 2) [19]. The bias domains assessed included (1) bias arising from the randomization process, (2) bias due to deviations from intended interventions, (3) bias due to missing outcome data, (4) bias in the measurement of the outcome, and (5) bias in the selection of the reported result. The overall risk of bias for each study was marked as (1) “low risk of bias” if all domains were judged to be at low risk of bias, (2) “some concerns” if at least one domain was judged to raise some concerns but not to be at high risk of bias for any domain, or (3) “high risk of bias” if any domain was judged to be at a high risk of bias. Disagreements between the mentioned researchers regarding the risk of bias were resolved through consensus or by a third researcher (OJS). The Covidence tool was used to assess the risk of bias.

### Data Synthesis (Statistical Methods)

The general strategy for data synthesis was to perform a quantitative synthesis (meta-analysis). Heterogeneity (*I*^²^) values were judged as follows: 0%-40% may represent little heterogeneity, 30%-60% may represent moderate heterogeneity, 50%-90% may represent substantial heterogeneity, and 75%-100% may represent considerable heterogeneity. Heterogeneity, which is the percentage of variation across studies owing to heterogeneity rather than chance, was evaluated for clinical, methodological, and statistical heterogeneity [19].

### Quantitative Synthesis

We expected clinical and methodological heterogeneity in the pooled studies; therefore, we applied a random effects model to obtain the overall effect size estimate. The inverse-variance method was used to perform the meta-analysis. Larger studies with less variance were given more weight in the meta-analysis owing to more precise effect size estimates than smaller studies.

### Continuous Outcome Measures

The standardized mean difference (SMD) effect size was calculated for the primary outcome using the Hedges g formula. Different assessment tools were used to calculate the effect size of the primary outcome in each study. Therefore, SMD was statistically suitable for estimating the effect size for each study. Forest plots were used to present study-specific effect sizes and overall effect sizes, including 95% CIs. Furthermore, we calculated the *I^²^* statistic to quantify heterogeneity and the *χ*² statistic to test for heterogeneity (*P*≤.10 significance level).

For the secondary outcomes—patient satisfaction and working alliance—the mean difference (MD) effect size was calculated as these secondary outcomes were assessed using a single standardized tool (CSQ-8 and WAI-C). Therefore, standardization was not needed to calculate the effect size across studies. The same statistical approach used for the primary outcome was applied to the secondary outcomes of patient satisfaction and working alliance.

Postintervention data (sample size, mean, and SD) for each treatment modality (in person and telemedicine) were used to calculate the effect size of the continuous outcome measures (efficacy, satisfaction, and alliance), which is considered a valid approach [22].

### Dichotomous Outcome Measures

The risk ratio effect size and its 95% CI was calculated for the secondary outcome attrition rate. A forest plot was created to present the effect size for each study and the overall effect size for pooled analysis and was supplemented with *I*² and *χ*² statistics.

### Additional Primary Outcome Analyses (Investigating Heterogeneity)

#### Moderator Analysis

For the primary outcome, subgroup analyses for different patient groups were performed based on (1) participant diagnosis, as specified in the eligibility criteria; (2) age; (3) length of treatment course or program; and (4) therapy type.

The year of study publication was evaluated through a meta-regression, and the *P* value for the regression was computed (*P*≤.05 significance level).

#### Sensitivity Analysis

A sensitivity analysis was also performed to determine the robustness of the meta-analysis and included (1) sensitivity testing for only high-quality trials and (2) testing for whether the findings were sensitive to random effects or fixed effects models.

### Meta-Bias

Publication bias was assessed visually using a funnel plot and tested statistically using the Egger test [19,23].

### Certainty of the Evidence

The GRADE (Grading of Recommendations Assessment, Development, and Evaluation) approach as recommended by the Cochrane Collaboration was used to assess the certainty (confidence) of the evidence [19,24]. The certainty of the evidence for the primary outcome was evaluated for 5 domains and included an evaluation of the risk of bias (Rob 2), inconsistency, indirectness, imprecision, and publication bias. Each domain was graded as having a “serious,” “very serious,” or “not serious” impact on the certainty of the evidence and was downgraded with 1 level, 2 levels, or no downgrading, respectively. The GRADE approach was conducted using GRADEpro GDT software [25]. Two independent authors performed the GRADE approach (AAS and OJS), and a third author (SA) was consulted when disagreements occurred. The certainty of the evidence will be presented in the GRADE summary of the findings table.

### Ethical Considerations and Dissemination

Ethical approval was not required for this systematic review and meta-analysis. The data sets (extraction) are deposited in the Zenodo repository (DOI:10.5281/zenodo.7339263). This study will be disseminated at scientific conferences.

## Results

### Study Selection

**Figure 1:**
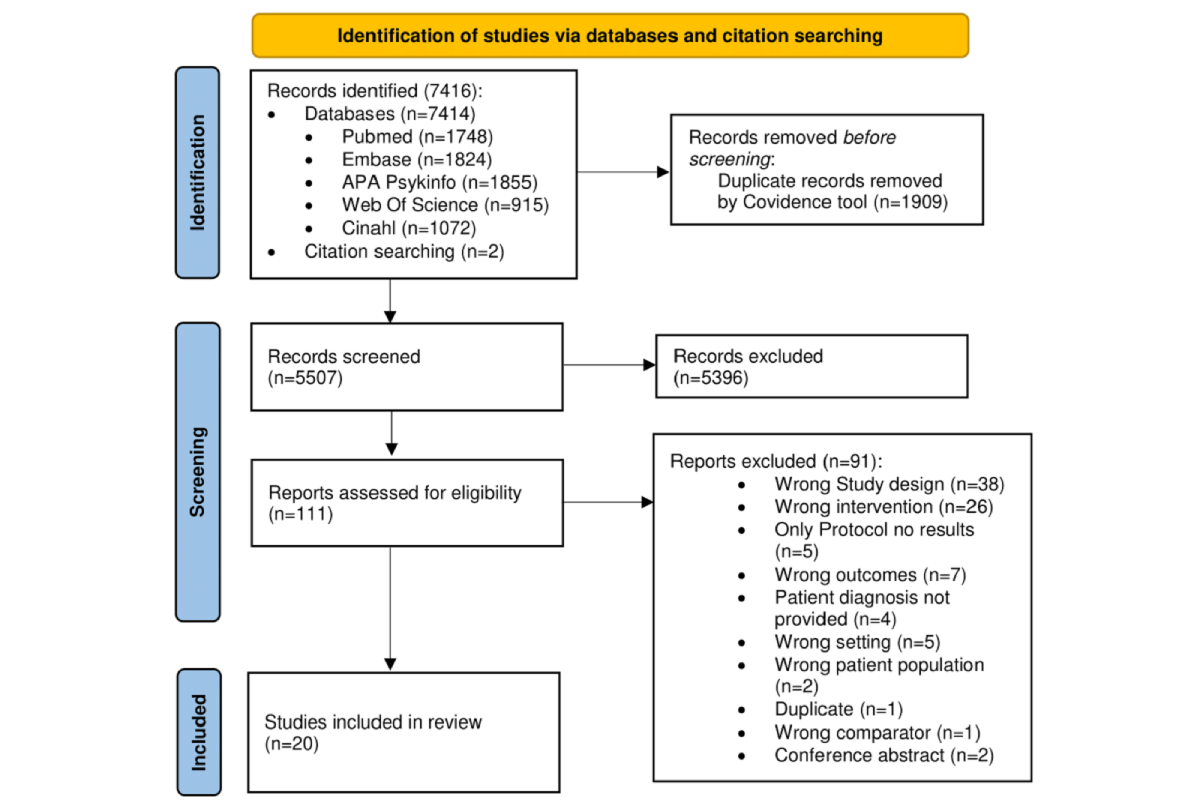
PRISMA (Preferred Reporting Items for Systematic Reviews and Meta-Analyses) flow diagram across all stages of article identification.

This study focused on outpatient psychiatric treatment conducted via telemedicine (video) or in person for PTSD, mood disorders, and anxiety disorders. The PRISMA flow diagram shows all stages of the article identification, screening, inclusion, and exclusion processes (Figure 1). Searches generated 7414 records. Two records were identified through manual literature search and reference list scanning. After removing the duplicates and applying inclusion and exclusion criteria to the titles and abstracts, 111 reports emerged as candidates for full-text review. A total of 20 studies were included in the final review. Cohen κ coefficient indicated fair interrater reliability for the title and abstract screening process (AAS and AA: Cohen κ=0.27), whereas it was moderate for the full-text review process (AAS and SFA: Cohen κ=0.52; AAS and JS: Cohen κ=0.5).

### Study Characteristics

#### Overview

Table 1 presents the descriptive characteristics of the 20 included studies.

**Table 1:** Overview of included studies.

Study, year	Sample size, n	Diagnosis	Age (years), mean (SD)	Female, n (%)	Intervention type	Number of sessions (interval in weeks)	Outcome or outcomes of interest (assessment instrument)	Bias (RoB 2^a^) assessed for primary outcome
Peterson et al [26], 2022	120	PTSD^b^	39.95 (10.36)	14 (11)	Cognitive processing therapy	12 (2 sessions per week)	Psychopathology (PCL-5^c^) Attrition	High risk
Acierno et al [27], 2021	136	PTSD	43.4 (11.5)	136 (100)	Prolonged exposure	14 (1)	Psychopathology (PCL-5) Attrition	High risk
Liu et al [28], 2020	207	PTSD	48.4 (14.1)	47 (23)	Cognitive processing therapy	12 (1)	Psychopathology (CAPS^d^) Attrition	Some concerns
Morland et al [29], 2020	175	PTSD	46.5 (14.11)	43 (24)	Prolonged exposure	6-15 (1)	Psychopathology (CAPS-5) Attrition	High risk
Watts et al [30], 2020	115	GAD^e^	41 (15.7)	95 (83)	Cognitive behavioral therapy	15 (1)	Psychopathology (ADIS^f^-IV) Working alliance (WAI-C^g^) Attrition	High risk
Haghnia et al [31], 2019	71	PTSD	Range: 45-60 year	0 (0)	Case management	9 (1)	Attrition	High risk
Acierno et al [32], 2016	265	PTSD	45.6 (14.9)	16 (6)	Behavioral activation	8 (1)	Psychopathology (PCL-Military version) Attrition	Some concerns
Hungerbuehler et al [33], 2016	107	Depression	35.64 (8.33)	76 (71)	Case management	5 (4)	Psychopathology (HDRS^h^-17) Satisfaction (CSQ^i^-8) Working alliance (WAI-C) Attrition	Some concerns
Luxton et al [34], 2016	121	Depression	Range: 19-65 year	22 (18)	Behavioral activation	8 (1)	Psychopathology (BDI^j^-II) Satisfaction (CSQ-8) Attrition	Low risk
Maieritsch et al [35], 2016	90	PTSD	30.93 (6.05)	6 (7)	Cognitive processing therapy	A minimum of 10 (1)	Psychopathology (CAPS) Working alliance (WAI-C) Attrition	Some concerns
Egede et al [36], 2015	241	Depression	63.9 (5.1)	5 (2)	Behavioral activation	8 (1)	Psychopathology (BDI) Attrition	Some concerns
Morland et al [37], 2015	124	PTSD	46.4 (11.9)	100 (100)	Cognitive processing therapy	12 (1)	Psychopathology (CAPS) Working alliance (WAI-C) Attrition	Low risk
Yuen et al [38], 2015	52	PTSD	43.98 (15.18)	1 (2)	Prolonged exposure	8-12 (1)	Psychopathology (CAPS) Attrition	Low risk
Choi et al [39], 2014	85	Depression	65.21 (9.22)	66 (78)	Case management	6	Psychopathology (HAM-Dh) Attrition	High risk
Stubbings et al [40], 2013	26	Mixed Diagnosis^k^	20 (11)	15 (58)	Cognitive behavioral therapy	12 (1)	Psychopathology (DASS^l^ subscales) Working alliance (WAI-C) Satisfaction (CSQ-8) Attrition	High risk
Chong et al [41], 2012	167	Depression	N/A^m^	148 (89)	Case management	6 (4)	Psychopathology (PHQ^n^-9) Working alliance (WAI-C) Attrition	Some concerns
O’Reilly et al [42], 2007	495	Mixed Diagnosis	Range: 18-65 year	312 (63)	Case management	Up to 4 (4)	Psychopathology (GSI^o^) Satisfaction (CSQ-8) Attrition	High risk
De Las Cuevas et al [43], 2006	140	Mixed Diagnosis	Range: 25-65 year	93 (66)	Cognitive behavioral therapy	8 (3)	Psychopathology (SCL^p^-90R) Attrition	Some concerns
Ruskin et al [44], 2004	119	Depression	49.7 (12.8)	14 (12)	Case management	8 (up to 7)	Attrition	High risk
Bishop et al [45], 2002	24	Mixed Diagnosis	Range: 18-75 year	17 (71)	Case management	8	Satisfaction (CSQ-8) Attrition	Some concerns

^a^RoB 2: revised Cochrane risk-of-bias tool for randomized trials.

^b^PTSD: posttraumatic stress disorder.

^c^PCL: posttraumatic stress disorder checklist.

^d^CAPS: Clinician-Administered Posttraumatic Stress Disorder Scale.

^e^GAD: general anxiety disorder.

^f^ADIS: anxiety disorders interview schedule.

^g^WAI-C: Working Alliance Inventory-Client version.

^h^HDRS or HAM-D: Hamilton Depression Rating Scale.

^i^CSQ: Client Satisfaction Questionnaire.

^j^BDI: Beck Depression Inventory.

^k^The 4 studies with “mixed diagnosis” included mainly patients with PTSD and depressive disorders.

^l^DASS: Depression Anxiety Stress Scales.

^m^N/A: not applicable.

^n^PHQ: Patient Health Questionnaire.

^o^GSI: Global Severity Index.

^p^SCL: symptom checklist.

#### Demographics

The number of participants included in each study ranged from 24 to 495 (mean 144, SD 101.40). Out of 20 studies, 14 (70%) studies reported mean ages ranging from 20 to 65 years (mean 44.32, SD 11.19), 5 (25%) studies reported different ranges and percentages of age, and 1 (5%) study did not provide information about participant age. Most studies included a mix of males and females (17/20, 85%); 10% (2/20) of studies consisted of only females, and 5% (1/20) of only males. The diagnoses included PTSD (9/20, 45% studies), depressive disorders (6/20, 30% studies), a mix of different diagnoses (4/20, 20% studies), and general anxiety disorder (1/20, 5% studies).

#### Interventions

A range of treatment interventions via telemedicine and in person were offered, including prolonged exposure (3/20, 15% studies), cognitive processing therapy (4/20, 20% studies), behavioral activation treatment (3/20, 15% studies), cognitive behavioral therapy (3/20, 15% studies), and case management (7/20, 35% studies). The overall number of sessions varied between 4 and 15, with a median of around 8 sessions.

#### Outcomes

This study had 4 outcomes of interest: treatment efficacy, working alliance, treatment satisfaction, and attrition rates. Attrition rates were the only outcome reported in every study (20/20, 100%), whereas efficacy was measured in 85% (17/20) of studies. Out of 20 studies, 6 (30%) studies measured treatment satisfaction using the CSQ-8, and 6 (30%) studies measured the working alliance using a version of the WAI-C.

### Treatment Efficacy (Psychopathology)

#### Overview

**Figure 2:**
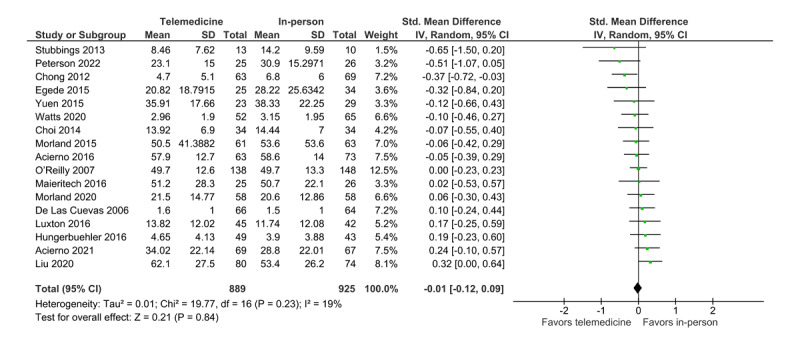
Forest plot (treatment efficacy) [26-30,32-43].

Data from 17 RCTs were pooled in the random-effect meta-analysis to examine the efficacy of treatment delivered via telemedicine and in person (Figure 2).

The effect size for each study was calculated and pooled. A negative effect size favors telemedicine treatment, whereas a positive effect size favors in-person treatment. The analysis shows that the CI for the overall estimated effect size include 0 (SMD=−0.01, 95% CI −0.12 to 0.09; *P*=.84; *I*^2^=19%, 17 trials, n=1814). Therefore, the result indicates no statistical difference in the treatment effect between the in-person and telemedicine modalities. The estimated total heterogeneity or *I*^2^ was 19%, indicating little heterogeneity. The 3 nonpooled studies, which did not provide enough information to be included in the quantitative analysis, reported the same results, that is, no difference between in-person and telemedicine treatment regarding efficacy [31,44,45].

#### Meta-Bias

**Figure 3:**
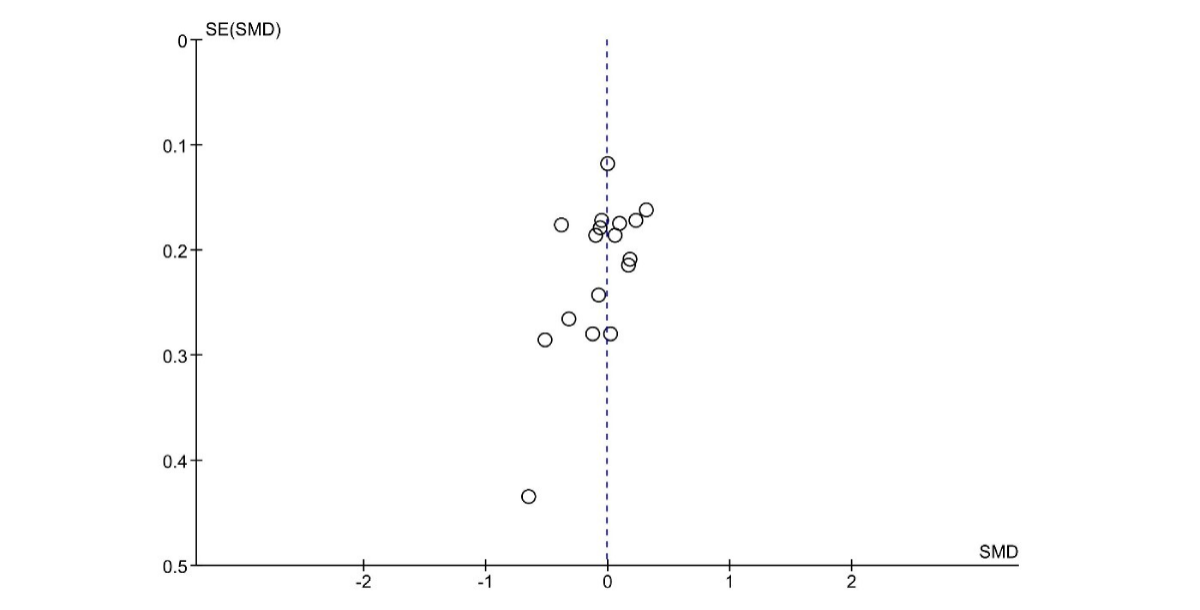
Funnel plot (efficacy). SMD: standardized mean difference.

Publication bias was visually evaluated using a funnel plot (Figure 3). The funnel plot shows symmetry, indicating no risk of publication bias. The risk of publication bias was also evaluated statistically using the Egger test, which did not reveal the presence of funnel plot asymmetry (intercept=−1.718, 95% CI −3.57 to −1.818, t_15_=−1.818; *P*=.09, 17 trials, n=1814).

#### Certainty of the Evidence

**Figure 4:**
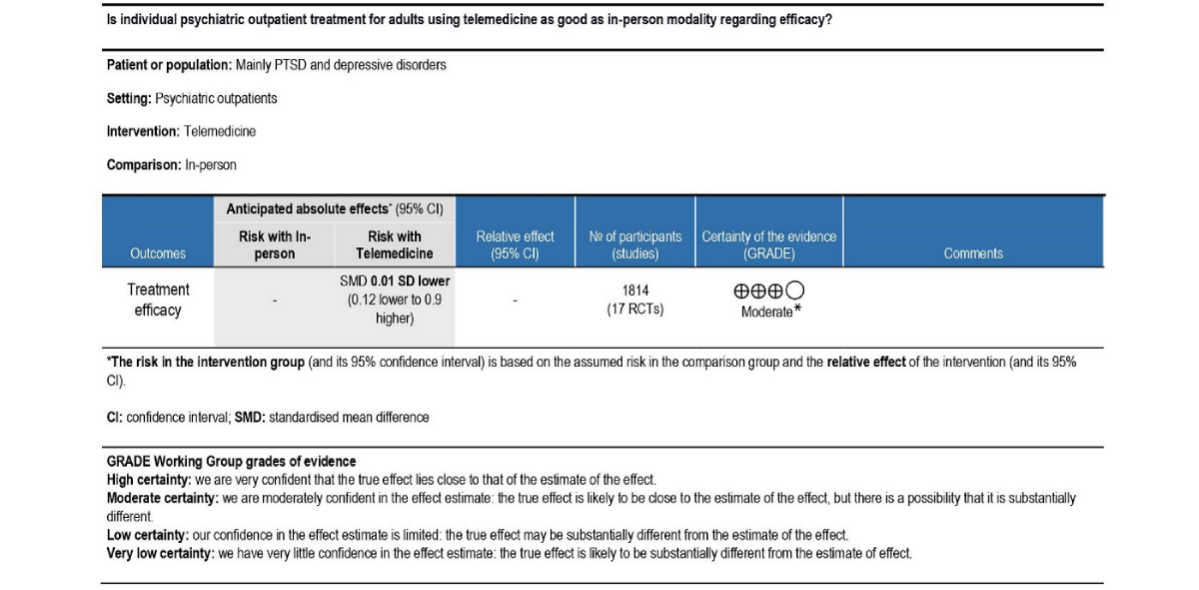
GRADE (Grading of Recommendations Assessment, Development, and Evaluation) summary of findings (treatment efficacy). *Evidence limited due to risk of bias.

The certainty of the evidence was rated as moderate according to GRADE (Figure 4). The downgrading was due to the risk of bias in multiple trials (poor reporting of how studies were planned and conducted). Of the 17 included trials, only 3 (18%) studies were rated as having a low risk of bias, 7 (41%) studies were rated as having some concerns of bias, and 7 (41%) studies were rated as having a high risk of bias.

#### Additional Primary Outcome Analyses (Investigating Heterogeneity)

##### Moderator Analyses

Moderator analyses were performed on 5 moderators: 4 performed in the subgroup analyses (age, diagnosis, number of sessions, and type of treatment) and 1 performed in a meta-regression analysis (publication year). The subgroup analyses presented in Table 2 indicate no statistical differences between the aggregated subgroups for any potential moderators. Most subgroup analyses showed little to moderate heterogeneity. In general, the results of the subgroup analyses must be interpreted with caution because of the small number of trials (<10) included in each subgroup analysis.

The meta-regression analysis included 17 trials and revealed no association between the estimated effect size and year of publication (*F*_1,15_=0.355; *P*=.56; *I*^2^=19.84, 17 trials, n=1814).

**Table 2:** Subgroup analyses.

Moderator			Included studies, n		SMD^a^ (95% CI)		*I* ^2^		*χ*^2^ (*df*)		Sample size, n		*P* value		*P*_subgroup_ value	
**Age**																.87
	Age (range values: 18-75 years)	4		–0.03 (0.25 to 0.18)		0.43		5.3 (3)		635		.15				
	Mean age (20-44 years)	7		–0.04 (–0.25 to 0.17)		0.29		8.5 (6)		522		.20				
	Mean age (45-65 years)	6		0.02 (–0.14 to 0.19)		0.12		5.7 (5)		657		.34				
**Diagnosis**																.60
	Mixed	3		–0.01 (–0.25 to 0.22)		0.22		2.6 (2)		439		.28				
	PTSD^b^	8		0.04 (–0.12 to 0.20)		0.21		8.9 (7)		820		.26				
	Depression	5		–0.08 (–0.33 to 0.16)		0.39		6.5 (4)		438		.16				
**Number of sessions**																.72
	>8	9		0.00 (–0.17 to 0.18)		0.32		11.7 (8)		824		.16				
	≤8	8		–0.04 (–0.17 to 0.10)		0.08		7.6 (7)		990		.37				
**Treatment**																.82
	Prolonged exposure	3		0.11 (–0.12 to 0.34)		0		1.3 (2)		304		.54				
	Cognitive Processing Therapy	4		–0.01 (–0.34 to 0.31)		0.57		7.0 (3)		380		.07				
	Behavioral activation	3		–0.04 (–0.28 to 0,20)		0.03		2.1 (2)		282		.36				
	Cognitive Behavioral Therapy	3		–0.07 (–0.36 to 0.23)		0.26		2.7 (2)		270		.26				
	Case Management	4		–0.07 (–0.29 to 0.15)		0.38		4.8 (3)		578		.19				

^a^SMD: standardized mean difference.

^b^PTSD: posttraumatic stress disorder.

##### Sensitivity Analyses

Sensitivity analyses were performed to determine the robustness of the meta-analysis. It included (1) removing low-quality studies (ie, studies rated as “some concerns” and “high concerns” assessed using RoB 2) and (2) testing whether the findings are sensitive to fixed effects models. Three studies, judged to be high-quality studies, were pooled together and yielded results supporting the robustness of the meta-analysis, that is, no difference in the efficacy between in-person and telemedicine modalities (SMD=0.00, 95% CI −0.24 to 0.25; *P*=.98; *I*^2^*=*0%, 3 trials, n=263). The overall estimated effect size was not sensitive to the fixed effects model (SMD=0.00, 95% CI −0.10 to 0.09; *P*=.92; *I*^2^=19%, 17 trials, n=1814), further supporting the robustness of the meta-analysis.

### Patient Satisfaction

**Figure 5:**
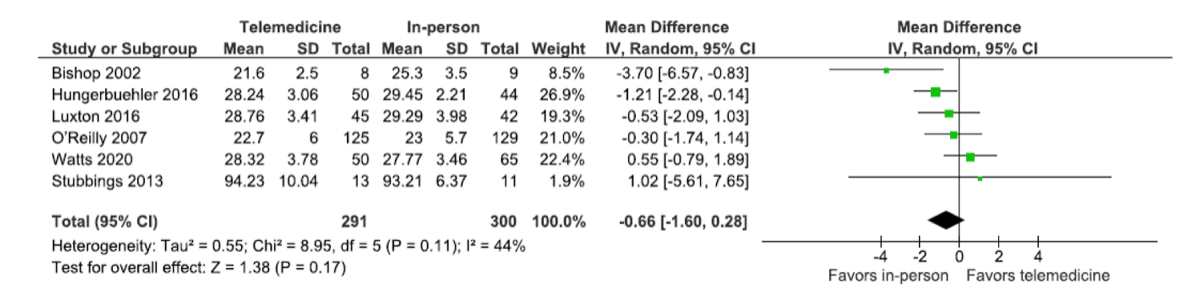
Forest plot (patient satisfaction) [30,33,34,40,42,45].

Patient satisfaction was assessed using CSQ-8, which was used in 6 trials. The forest plot shows the overall estimated effect size for the pooled trials (Figure 5). The analysis shows that the overall estimated effect size include 0 (MD=−0.66, 95% CI −1.60 to 0.28; *P*=.17; *I*^2^=44%, 6 trials, n=591). Therefore, the results indicate no statistical difference in patient satisfaction between the in-person and telemedicine treatment modalities. The estimated total heterogeneity for the analysis is 44%, indicating moderate heterogeneity.

### Working Alliance

**Figure 6:**
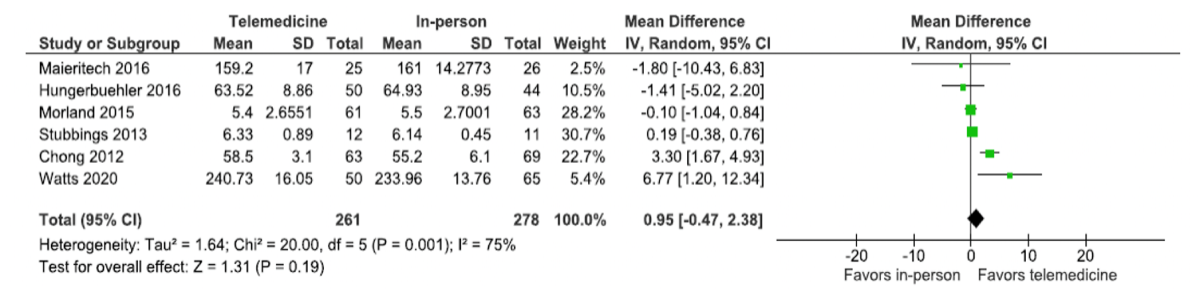
Forest plot (working alliance) [30,33,35,37,40].

The working alliance, as rated by patients, was assessed using WAI-C, which was applied in 6 trials. The forest plot shows the overall estimated effect size for the pooled trials (Figure 6). The analysis shows that the overall estimated effect size include 0 (MD=0.95, 95% CI −0.47 to 2.38; *P*=.19; *I*^2^=75%, 6 trials, n=539). Thus, the results indicate no statistical difference in the working alliance between the in-person and telemedicine treatment modalities, and the levels of the working alliance are comparable for the 2 treatment modalities. However, the estimated total heterogeneity for the analysis is 75%, indicating substantial to considerable heterogeneity and is statistically significant (*χ^2^*_5_*=*20; *P=*.001).

### Attrition Rate

**Figure 7:**
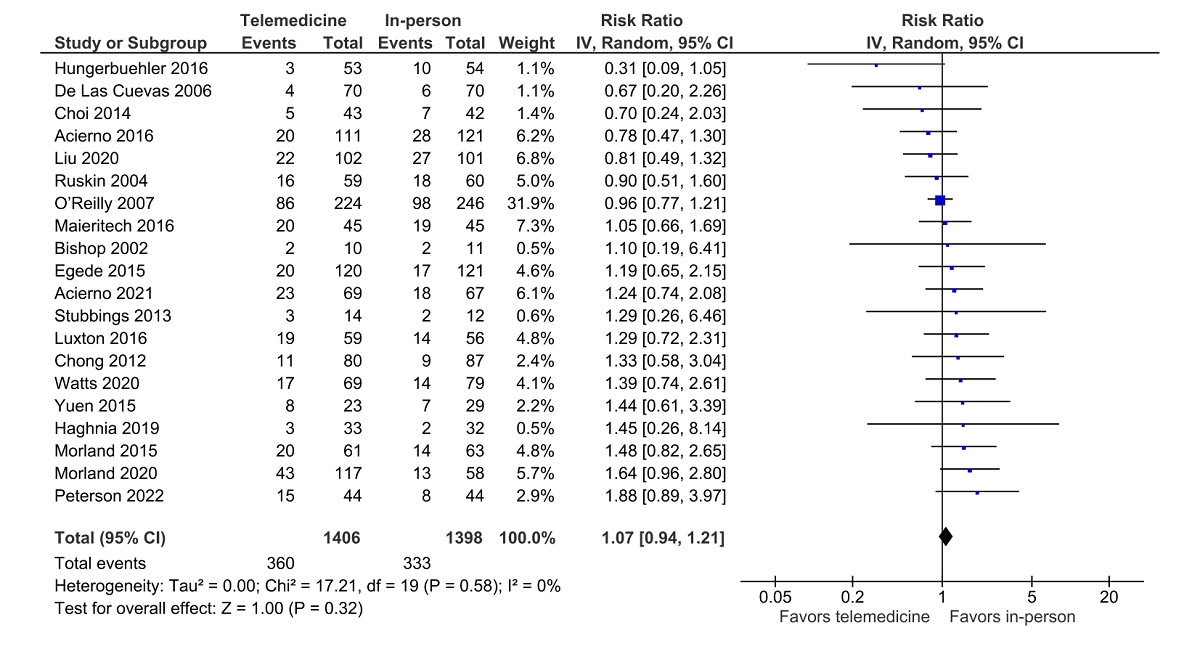
Forest plot (attrition rate).

All trials included in the meta-analysis (N=20) reported data on attrition rates. The number of attrition events for each modality was either directly extracted from the studies or calculated by subtracting the number of completers from the number of those randomized to a treatment modality. The forest plot shows the overall estimated effect size for the pooled trials assessing the attrition ratio between the modalities (Figure 7). The analysis shows that the overall estimated effect size include 1; therefore, the results indicate no statistical difference in patient attrition rates between the in-person and telemedicine treatment modalities (risk ratio 1.07, 95% CI 0.94-1.21; *P*=.32; *I*^2^=0%, 20 trials, n=2804). The estimated total heterogeneity for the analysis is 0%, indicating no heterogeneity. The funnel plot and the Egger test was conducted and did not reveal the presence of asymmetry, indicating no risk of publication bias. Post hoc analysis was conducted to evaluate attrition outcomes based on diagnosis (PTSD and Depression), which did not influence the overall findings.

## Discussion

### Principal Findings

The primary objective of this systematic review and meta-analysis was to evaluate whether individual psychiatric outpatient treatment for adults with PTSD, mood disorders, and anxiety disorders using telemedicine is comparable with in-person treatment. Second, the study evaluated whether patient satisfaction, working alliance using standard measures, and attrition rates were comparable between telemedicine and in-person treatment modalities. A comprehensive literature search for RCTs comparing telemedicine with in-person modalities was conducted. Using stringent eligibility criteria*,* 20 RCTs that met prespecified eligibility criteria were identified [14]. Overall, the study results indicate that treatment delivered through telemedicine is comparable with in-person treatment modality regarding treatment efficacy, patient satisfaction, working alliance, and attrition rate.

The primary outcome evaluated in this systematic review was efficacy and included 17 trials assessing treatment effects between the telemedicine and in-person treatment modalities. The results did not indicate a risk of publication bias. To test the robustness of the overall finding, that is, no difference in the treatment effect between telemedicine and in-person treatment modality regarding efficacy, prespecified sensitivity analyses were conducted, which supported the overall finding. Different moderators were tested for their potential influence on efficacy and included different age groups, diagnoses, number of sessions, types of psychotherapy, and publication year. None of the evaluated moderators affected the overall findings, strengthening the evidence for the equality between telemedicine and in-person treatment on various clinical and methodological characteristics. The results of this systematic review and meta-analysis are consistent with those of previously published meta-analyses, indicating the nonsuperiority of in person to telemedicine across psychiatric diagnoses [11-13]. However, when interpreting the results for the moderators in the subgroup analysis, it is important to note that relatively few studies (<10) were included in each moderator analysis, which is a considerable limitation [46]. The meta-analysis focused on PTSD, mood disorders, and anxiety disorders in which most trials included were focused on PTSD (n=8) and depression (n=5). There were no studies included that compared the treatment effects between telemedicine and in person for patients with personality disorders, social phobia, and agoraphobia. Further studies are required to determine the efficacy of psychiatric treatment using telemedicine for these disorders.

The secondary outcomes investigated between the 2 treatment modalities included patient satisfaction, working alliance, and attrition rates.

Satisfaction measurement was limited to CSQ-8, resulting in 6 RCTs in the meta-analysis. The results indicate that satisfaction between treatment modalities (in person and telemedicine) is comparable, and heterogeneity is low to moderate. To our knowledge, satisfaction has only been evaluated in a single meta-analysis by Hyler et al [47], who concluded the equivalence in satisfaction between telemedicine and in-person treatment modalities regarding psychiatric assessment. However, the authors also mention limitations with the study owing to the ad hoc and untested instruments applied for measuring satisfaction and pooling the satisfaction measure for both the patients and therapists in the same analysis. This meta-analysis on satisfaction was restricted to a single validated questionnaire (CSQ-8), strengthening the evidence for equivalence in treatment satisfaction between telemedicine and in-person modalities.

The assessment of working alliance was limited to alliance reported by patients (WAI-C), resulting in 6 RCTs in the meta-analysis. The results indicate that the working alliance between treatment modalities was comparable, although heterogeneity was substantial to considerable (*I*^2^=75%; *χ*^2^_5_=20; *P*=.001), weakening the finding. Further analyses investigating heterogeneity were not possible because of the small number of included studies applying WAI-C, and this was not prespecified in the protocol. A working alliance is considered an important factor in psychotherapy outcomes, and clinicians have shown some concern that the telemedicine format of treatment may negatively impact the working alliance [48,49]. Previous research on alliance in telemedicine interventions has shown varied results but generally shows that a good therapeutic alliance can be established in telemedicine interventions and is comparable with in-person interventions [50]. Although patients consistently rate working alliance as good in telemedicine interventions, therapists have shown a tendency to rate the alliance as lower than in-person interventions. Norwood et al [3] evaluated the working alliance in a noninferiority meta-analysis and found that alliance in telemedicine is inferior to in person. Although this study combined ratings of patient and therapist alliance, the authors acknowledged that this procedure may have reduced the overall levels of alliance in the telemedicine condition. To our knowledge, this meta-analysis is the first study to examine therapeutic alliance as rated only by the patients. The comparable levels of alliance reported by patients are further supported by the similar levels of attrition and patient satisfaction rates between the telemedicine and in-person interventions found in this meta-analysis. Future studies should aim to examine how the working alliance is established over time and its implications for outcomes when treatment is delivered through telemedicine.

The attrition rate was comparable between treatment modalities (telemedicine and in person) across diagnoses (PTSD and depression) and is consistent with the results published by Giovanetti et al [13]. The authors included 11 RCTs and found equivalent attrition rates between the telemedicine and in-person treatment modalities; however, the authors only assessed attrition rates in patients with depression. Thus, the telemedicine modality of psychiatric treatment did not appear to negatively impact sustained engagement when compared with treatment via the in-person modality.

### Study Limitations

First, the stringent eligibility criteria regarding population, intervention, control, and outcome measures limited the total number of studies available for analysis. Second, the inadequate and poor reporting of the included studies led to only 3 studies being evaluated as high-quality based on the RoB 2 criteria. Owing to the limited number of high-quality studies, the certainty of the evidence (according to GRADE) was rated as moderate, and a high certainty of the evidence was therefore not achievable for the efficacy outcome. Third, data were nonuniformly reported in a number of the included studies. For example, age was reported as ranges (percentages) in some studies, whereas other studies reported the mean age, making it challenging to aggregate subgroups for the moderator analysis. Finally, most studies did not provide separate results for male and female participants, so it was not possible to determine whether there was a differential effect of sex.

### Implications for Clinical Practice and Research

Rapid digitalization within mental health care is changing clinical practice. This study provides a comprehensive and up-to-date meta-analytic overview of the use of telemedicine in individual psychiatric treatment across a range of diagnoses and clinical outcomes. Evidence generated by this meta-analysis can guide clinical practice regarding which disorders can be effectively treated using individual psychiatric treatment conducted via telemedicine and which psychotherapy approaches are effective.

Importantly, the results of this review revealed a complete absence of high-quality studies examining the efficacy of psychiatric treatment via telemedicine for personality disorders and a range of anxiety disorders. This knowledge gap must be addressed in future studies. Future studies should also examine how to identify the most suitable treatment modality (telemedicine, in person, telephone, etc) for psychiatric patients, thereby matching patient needs to the treatment mode to optimize outcomes. Using individual patient data, meta-analysis within telemedicine applications could potentially address this challenge.

### Conclusions

In summary, the results of this meta-analysis indicate that psychiatric treatment via telemedicine for PTSD, mood disorders, and anxiety disorders were equivalent to in-person treatment in terms of treatment efficacy, satisfaction, and attrition rate. Although working alliance as rated by patients was also deemed to be comparable between the 2 modalities, heterogeneity in the analysis was substantial. Thus, there is a need for further high-quality controlled trials to fully understand the complex issue of working alliance for interventions conducted via telemedicine.

## Appendix

Multimedia Appendix 1 PRISMA (Preferred Reporting Items for Systematic Reviews and Meta-Analyses) checklist.

## Abbreviations

CSQ-8: Client Satisfaction Questionnaire-8
GRADE: Grading of Recommendations Assessment, Development, and Evaluation
MD: mean difference
MeSH: Medical Subject Headings
PRISMA: Preferred Reporting Items for Systematic Reviews and Meta-Analyses
PTSD: posttraumatic stress disorder
RCT: randomized controlled trial
RoB 2: revised Cochrane risk-of-bias tool for randomized trials
SMD: standardized mean difference
WAI-C: Working Alliance Inventory-Client version
