# Correction: Psychiatric Treatment Conducted via Telemedicine Versus In-Person Modality in Posttraumatic Stress Disorder, Mood Disorders, and Anxiety Disorders: Systematic Review and Meta-Analysis

**DOI:** 10.2196/52269

**Published:** 2023-09-18

**Authors:** Ali Abbas Shaker, Stephen F Austin, Ole Jakob Storebø, Julie Perrine Schaug, Alaa Ayad, John Aasted Sørensen, Kristine Tarp, Henrik Bechmann, Erik Simonsen

**Affiliations:** 1 Psychiatric Department, Region Zealand Psychiatry, Psychiatric Research Unit Slagelse Denmark; 2 Department of Clinical Medicine, University of Copenhagen Copenhagen Denmark; 3 Department of Psychology, University of Southern Denmark Faculty of Health Sciences Odense Denmark; 4 Department of Engineering Technology and Didactics, Research unit: AI, Mathematics and Software, Technical University of Denmark Ballerup Denmark; 5 Research Unit for Digital Psychiatry, Mental Health Services in the Region of Southern Denmark Odense Denmark; 6 Department of Clinical Research, University of Southern Denmark Odense Denmark; 7 Mental Health Services East, Copenhagen University Hospital – Psychiatry Region Zealand Roskilde Denmark

In “Psychiatric Treatment Conducted via Telemedicine Versus In-Person Modality in Posttraumatic Stress Disorder, Mood Disorders, and Anxiety Disorders: Systematic Review and Meta-Analysis” (JMIR Ment Health 2023;10:e44790), the authors noted one error.

In Figures 5 and 6, the labels on the forest plot, “Favors telemedicine” and “Favors in-person,” were swapped. In the revised figures, these labels are presented correctly, as follows:

**Figure 5:**
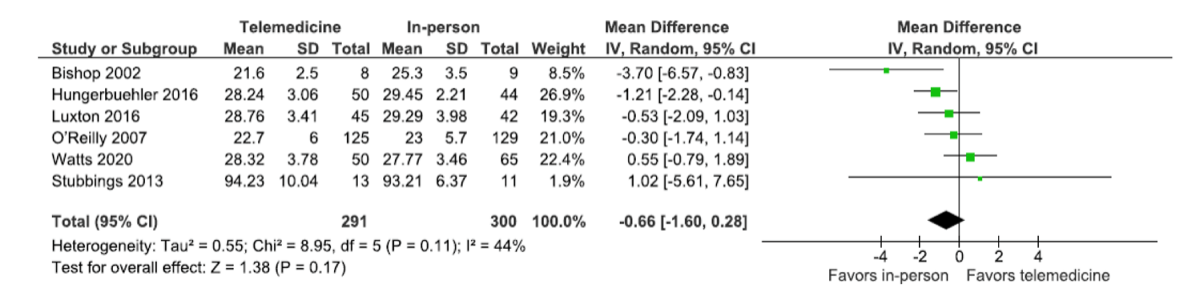
Forest plot (patient satisfaction).

**Figure 6:**
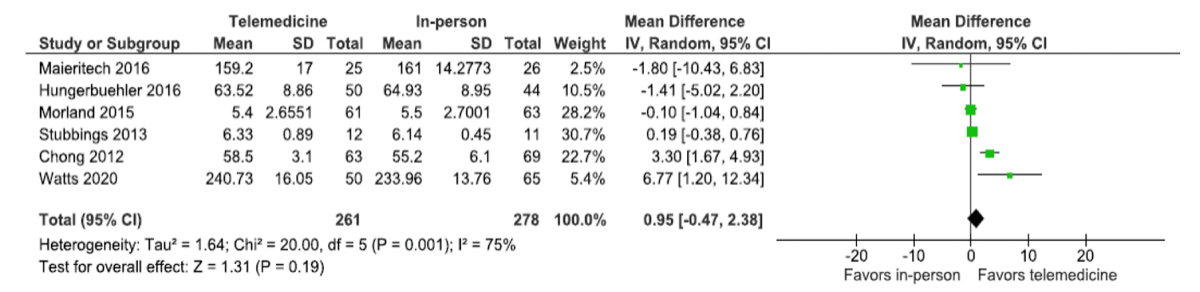
Forest plot (working alliance).

The correction will appear in the online version of the paper on the JMIR Publications website on September 18, 2023 together with the publication of this correction notice. Because this was made after submission to PubMed, PubMed Central, and other full-text repositories, the corrected article has also been resubmitted to those repositories.

